# In her own time

**Published:** 2018-11-12

**Authors:** Flora Jung

**Affiliations:** 1 University of Toronto, Ontario, Canada

**Figure:**
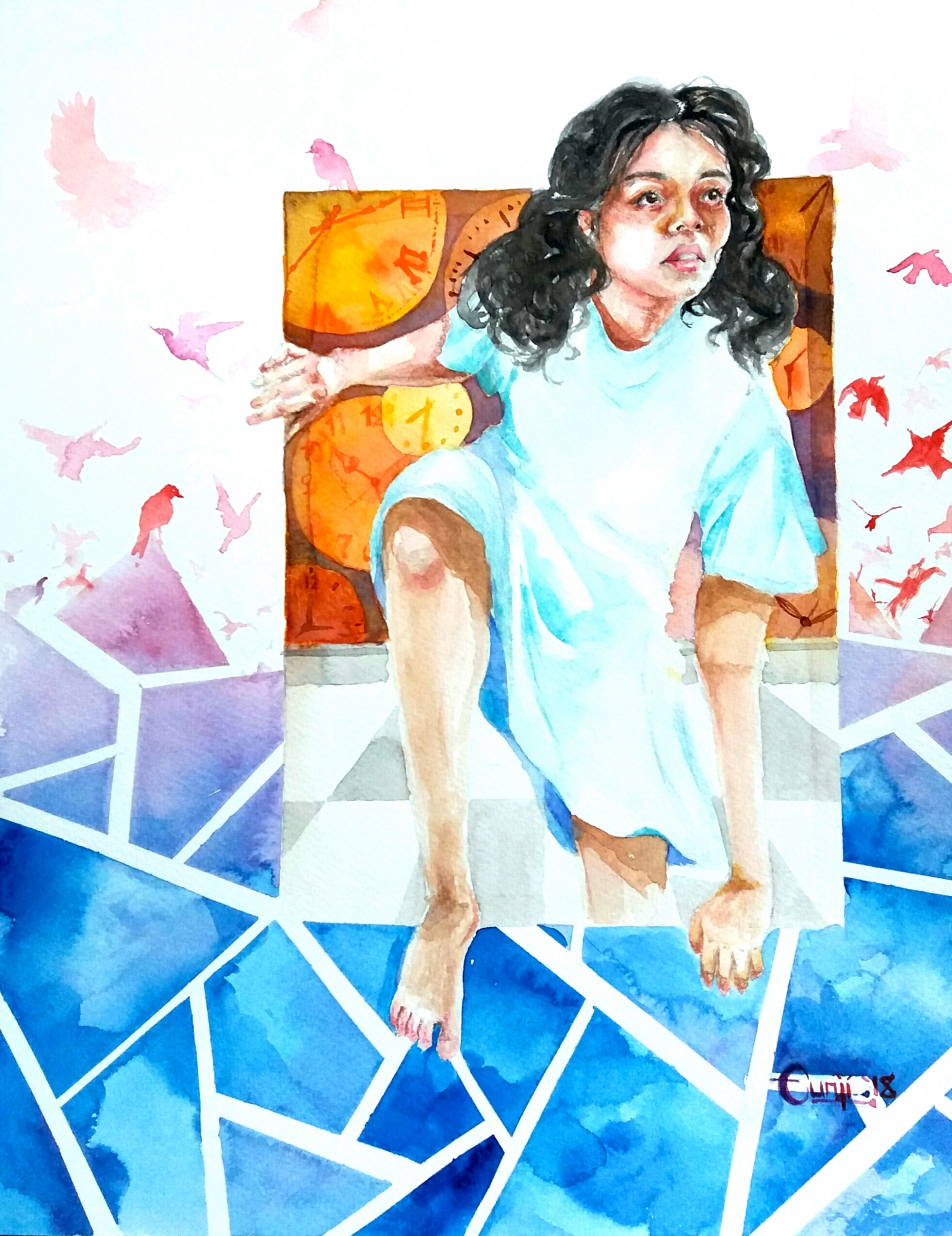


As I look back on my first year as a medical student, I am dazed by the whirlwind of emotions and experiences I met within this short time. One recurring theme emerged from my first experiences in clinic. Younger and healthier patients are often asked to be our bedside teachers to facilitate our learning, but consequently, I found it uncannily natural to see myself in them. Each patient and their tale of tenacity, misery, sanguinity, and bravery would leave me lost in thought for days to follow. *What if that were me?*

My art allows me to bring these thoughts to the tangible world and moves me to deeper introspection. In some of my first attempts to recreate an individual’s expression or the background which would complement their character, I found the challenge demanded observation and empathy that was much more than surface-deep. It stimulated me to practice visualizing the patient in a more holistic manner, not just as a person, but as also their emotions, choices, environment, past and future.

“In her own time” is one of these attempts to capture a story about a girl who I felt was unjustly young to be a patient. I tried to express my hope for her to slip free from the weight of time and fate to find solace. Although as a trainee, I wasn’t able to follow her for very long, this painting allowed me to explore her story further and find closure.

## About the author

Flora Jung is a hobby watercolour artist and a medical student at the University of Toronto. Rather than words, she enjoys painting as a platform to tell and remember stories from her personal life, and is inspired by her interests in medical education, the human experience, and the natural world.

